# Model Constrained by Visual Hierarchy Improves Prediction of Neural Responses to Natural Scenes

**DOI:** 10.1371/journal.pcbi.1004927

**Published:** 2016-06-27

**Authors:** Ján Antolík, Sonja B. Hofer, James A. Bednar, Thomas D. Mrsic-Flogel

**Affiliations:** 1 Unité de Neurosciences, Information et Complexité, CNRS UPR 3293, Gif-sur-Yvette, France; 2 Department of Neuroscience, Physiology and Pharmacology, University College London, London, United Kingdom; 3 Biozentrum, University of Basel, Basel, Switzerland; 4 Institute for Adaptive and Neural Computation, University of Edinburgh, Edinburgh, United Kingdom; University of Tübingen and Max Planck Institute for Biologial Cybernetics, GERMANY

## Abstract

Accurate estimation of neuronal receptive fields is essential for understanding sensory processing in the early visual system. Yet a full characterization of receptive fields is still incomplete, especially with regard to natural visual stimuli and in complete populations of cortical neurons. While previous work has incorporated known structural properties of the early visual system, such as lateral connectivity, or imposing simple-cell-like receptive field structure, no study has exploited the fact that nearby V1 neurons share common feed-forward input from thalamus and other upstream cortical neurons. We introduce a new method for estimating receptive fields simultaneously for a population of V1 neurons, using a model-based analysis incorporating knowledge of the feed-forward visual hierarchy. We assume that a population of V1 neurons shares a common pool of thalamic inputs, and consists of two layers of simple and complex-like V1 neurons. When fit to recordings of a local population of mouse layer 2/3 V1 neurons, our model offers an accurate description of their response to natural images and significant improvement of prediction power over the current state-of-the-art methods. We show that the responses of a large local population of V1 neurons with locally diverse receptive fields can be described with surprisingly limited number of thalamic inputs, consistent with recent experimental findings. Our structural model not only offers an improved functional characterization of V1 neurons, but also provides a framework for studying the relationship between connectivity and function in visual cortical areas.

## Author Summary

A key goal in sensory neuroscience is to understand the relationship between sensory stimuli and patterns of activity they elicit in networks of sensory neurons. Many models have been proposed in the past; however, these models have largely ignored the known architecture of primary visual cortex revealed in experimental studies, thus limiting their ability to accurately describe neural responses to sensory stimuli. Here we propose a model of primary visual cortex that takes into account the known architecture of visual cortex, specifically the fact that only a limited number of thalamic inputs with stereotypical receptive fields are shared within a local area of visual cortex, and the hierarchical progression from neurons with linear receptive fields (simple cells) to neurons with non-linear receptive fields (complex cells). We show that the proposed model outperforms state-of-the-art methods for receptive field estimation when fitted to two-photon calcium recordings of local populations of mouse V1 neurons responding to natural image stimuli. The model demonstrates how the diverse set of receptive fields in the local population of neurons can be constructed from a limited number (< 20) thalamic inputs.

## Introduction

The fundamental assumption underlying early sensory processing is that different external stimuli elicit distinct activity patterns that encode the content of the stimuli. Patterns of neuronal activity in early sensory areas of cortex are themselves a product of the network in which the neurons are embedded [1,2]. Understanding the relationship between stimuli and responses in a given neural population, and how these responses are created by the underlying neural circuits, is thus essential for explaining the role of these neurons in sensory processing [3].

A common approach for identifying stimulus-response functions is to present a large set of stimuli while recording the responses of individual neurons, and subsequently fit each neuron with a model. The accuracy of the model can be determined by comparing the predicted and actual activities in responses to a novel stimulus set. This data-driven approach to describing stimulus response functions (e.g. spatio-temporal response functions, STRFs) of neurons in the visual system has been refined over the last four decades. Initially, the filter functions of functionally linear neurons in the retina, lateral geniculate nucleus (LGN), or simple cells in primary visual cortex (V1) were obtained using artificial sets of stimuli, such as sparse noise or M-sequences [4–6]. More recently, studies advanced to describing the response functions of less linear neurons (complex cells in primary visual cortex, and neurons in V2) [7,8] while using stimuli more representative of the natural environment, such as sequences or movies of natural scenes [9–12]. However, even in V1, the modest response prediction accuracy from these models indicates that our current ability to characterize the stimulus response functions is incomplete [8,10,13].

Several major advances in the estimation of response functions have been introduced in recent years. Spike-triggered covariance (STC) [7] and multi-layer neural networks [13–15] made it possible to estimate the non-linear receptive fields (RFs) of complex cells. Most previous methods for estimating RFs in the early visual system have dealt with data from single cells independently [4–6,16,17]. The introduction of generalized linear models (GLMs) showed that incorporating information about the activity of nearby neurons a few milliseconds in the past can significantly improve predictive power [2], but this technique is restricted to a linear representation of the receptive field. More recently, usage of pre-defined banks of linear and non-linear filters to pre-process the visual input, and then using linear regression in this transformed input space to fit the model, has improved prediction accuracy [8,18]. However, no RF estimation method has taken advantage of the fact the RFs of a local population of neurons are constructed from a limited number of shared LGN inputs [19], which have stereotypical center-surround RF structure. The advent of two-photon calcium imaging makes it possible to record the activity from complete local populations of neurons [20,21], and thus allows estimation of a model containing these constraints.

Here we propose a new method for estimating RFs in V1—the Hierarchical Structural Model (HSM)—which assumes that a local neuronal population shares a limited number of afferent inputs from the LGN. The model explicitly incorporates hierarchical sub-cortical and cortical processing, whereby center-surround thalamo-cortical inputs are summed in the first layer of neurons, consisting of putative simple cells, followed by a second layer of neurons that sum inputs from simple cells to form both simple and complex-cell like RFs. The model takes advantage of the RF redundancies among nearby V1 neurons, by simultaneously fitting the entire local population of recorded neurons, and outperforms current state-of-the-art approaches to RF estimation when predicting neuronal activity measured with two-photon calcium imaging.

## Results

**Fig 1:**
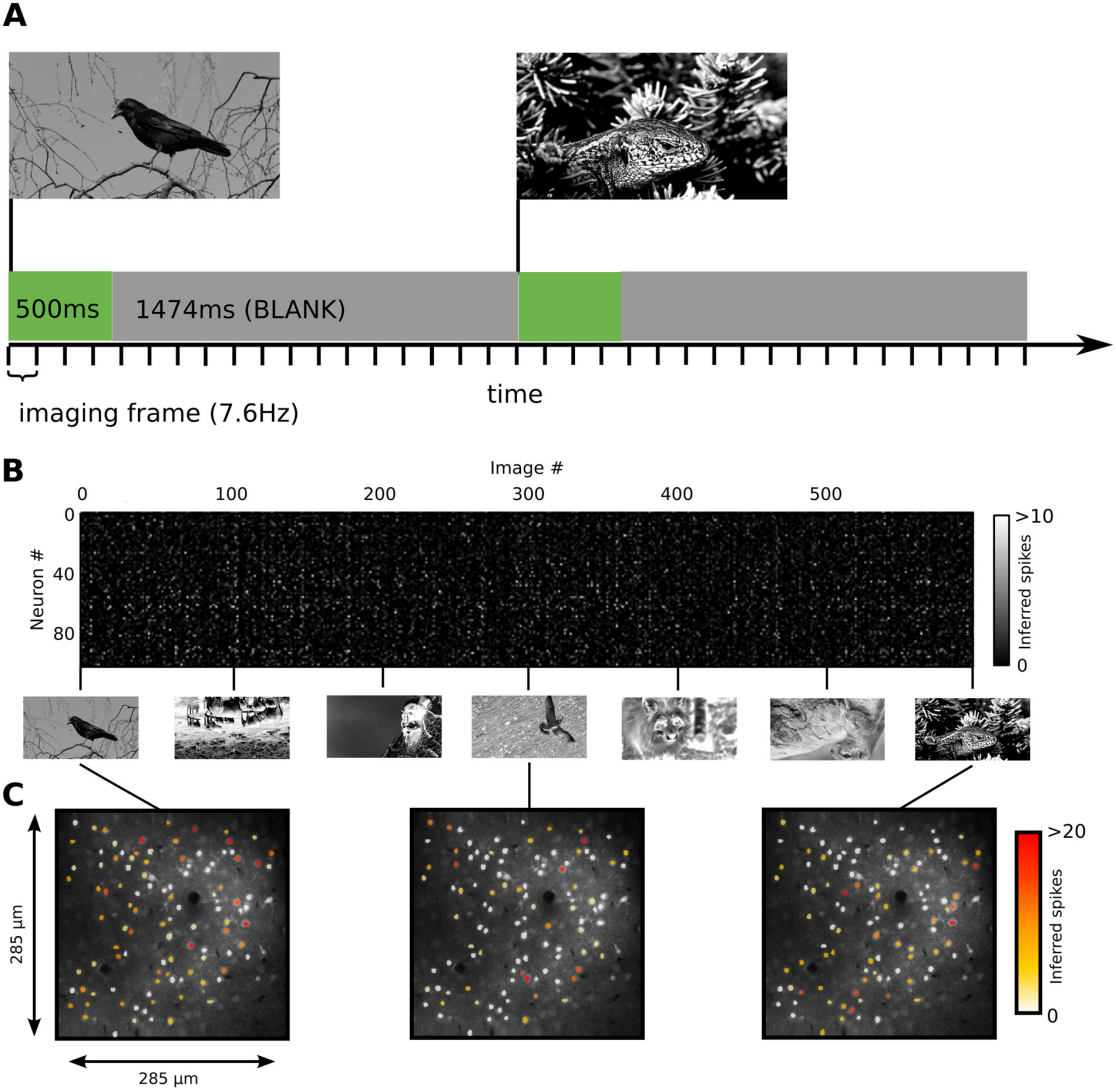
The stimulation protocol and population responses in L2/3 of mouse V1. (A) Natural images were presented for 500 ms, interleaved by 1474 ms periods of blank gray screens. (B) The responses (inferred spike rate) of 103 measured neurons to the first 600 of the 1800 images presented to the animal as a part of the training set (note that due to copyright restrictions the images presented during experiments were in this figure replaced with different equally pre-processed images which are under Creative Commons CC0 license). (C) Examples of spatial activity patterns to single natural images.

We measured neuronal responses with two-photon calcium imaging of local populations of mouse V1 neurons labeled with Oregon Green Bapta-1 AM (OGB-1), during presentation of a large set of full-field natural images (see Fig 1 and Materials and Methods). We recorded calcium signals in populations of layer 2/3 neurons in V1 (three regions from two mice) in response to unique set of 1260 to 1800 images. To obtain reliable responses we applied a stimulation protocol in which images were presented for 500 ms, interleaved with blank grey screens presented for 1474 ms. An extra 50 images were presented for 8–12 trials each, providing a set of neuronal responses used as a validation dataset. Calcium signals were converted into putative spikes using a fast non-negative de-convolution method [22]. Combined cell-attached recordings and calcium imaging were used to validate the spike inference method in a subset of neurons (53±6% single spikes and 95±2% of burst of 2 spikes detected per imaging frame; false positive rate 0.049±0.009 Hz [22,23]).

### Model-based RF estimation in mouse V1

**Fig 2:**
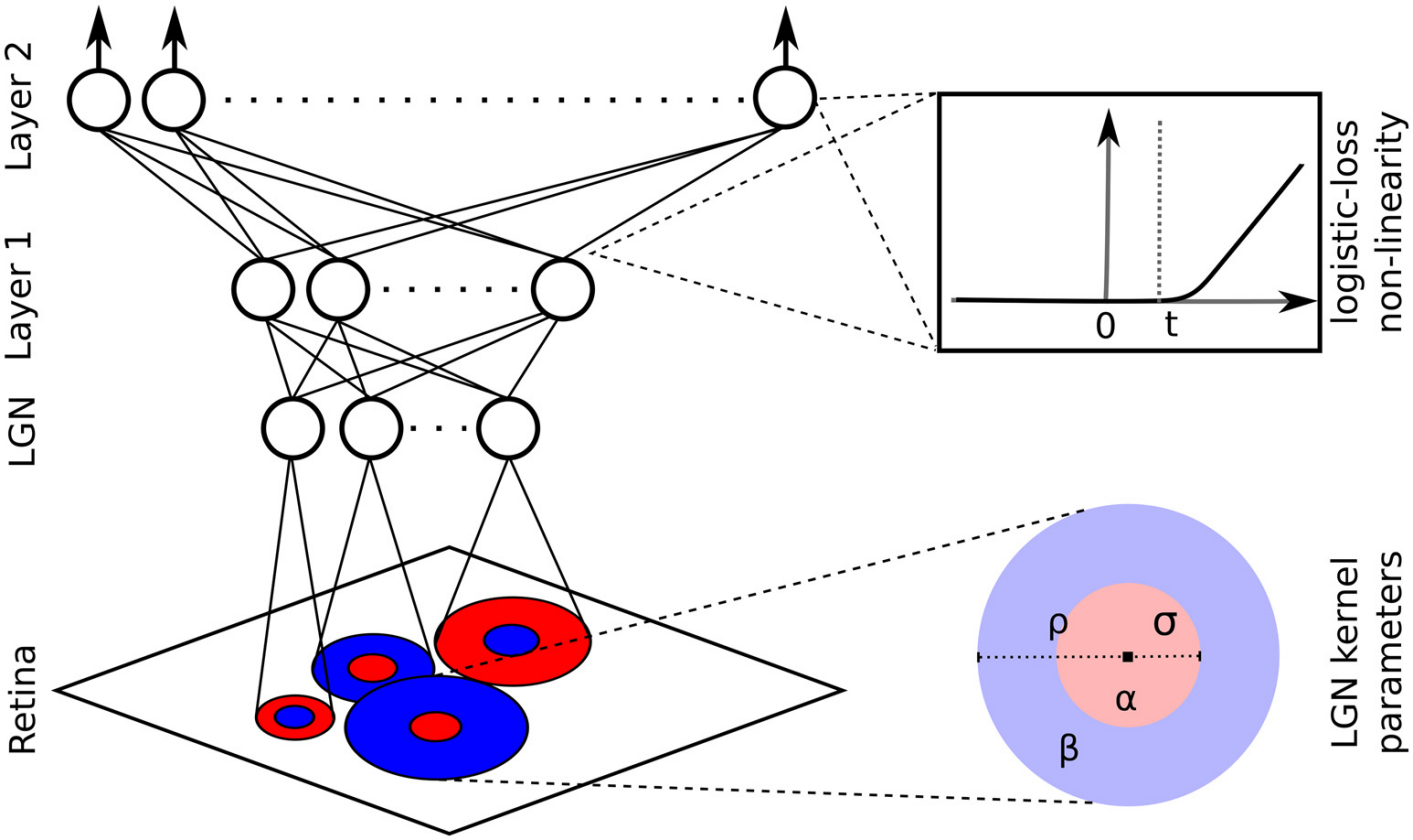
The architecture of the HSM. The model consists of a limited number of difference-of-Gaussian kernels, parameterized by the width and weight of the central and surrounding Gaussians, and the *x* and *y* coordinates of their center. This LGN layer is followed by two `cortical`layers of simple integrators with logistic-loss type transfer functions. The two layers are inter-connected by all-to-all connections and the first layer has all-to-all connections from the LGN units. Each unit in the two ‘cortical’ layers is parameterized by the set of incoming weights and the threshold of its logistic-loss transfer function. The log-loss function approximates a linear function with the slope of 1 as (*x* − *t*)→∞.

Our model-based approach to RF estimation is inspired by the anatomical and functional organization of mammalian V1 (see Fig 2 and Materials and Methods). It is based on the following basic assumptions: LGN units can be well described as difference-of-Gaussian functions [24]; the local population of V1 neurons shares input from limited number of such LGN units [19,25]; simple cells can be constructed by summing several RFs of LGN neurons [26,27]; complex cells can be constructed by summing inputs from the local population of simple cells that are selective to the same orientation but different RF phases [27].

The HSM consists of 3 layers of units: the first layer consists of linear kernels of LGN units that are modeled as 2D difference-of-Gaussians functions (see Fig 2). Units in the second layer sum the responses of LGN-like linear units, and pass on the resulting potential via a logistic-loss non-linearity. In this way units in the second layer construct oriented RFs through feed-forward summation of thalamocortical inputs [27]. Linear summation coupled with logistic-loss non-linearity is repeated again in the third layer, which enables construction of RFs that are tuned to orientation but can be insensitive to spatial phase (i.e. units resembling complex cells). Moreover, this approach also allows the generation of RFs that do not conform to the standard idealized models of either simple or complex cells, including, for example, models of cells that are selective to two orthogonal orientations. The HSM therefore leverages the assumed local connectivity in V1, thus potentially improving fits on limited data, while fitting RFs that do not conform to the idealized models of V1 neurons, a requirement that may be important for capturing the full response variability of V1 neurons.

**Fig 3:**
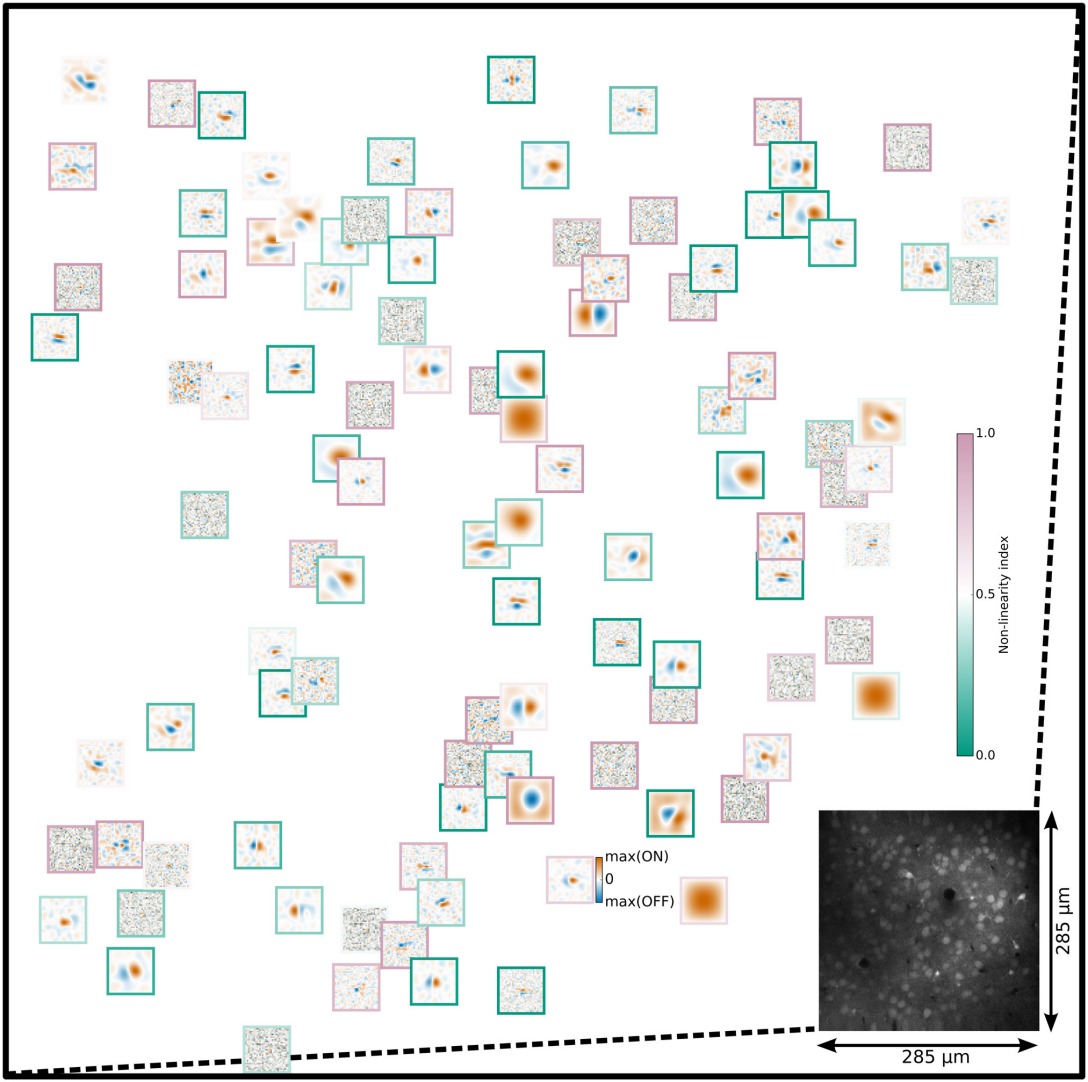
The distribution of linear RFs in the cortical space obtained from the first recorded region. Each linear RF is centered on the corresponding neuron’s cell body in the cortical region where recordings took place (displayed in lower right corner). The color of the frame indicates the NLI of the given neuron (blue-green scale bar). A wide variety of linear RFs were observed, and no specific ordering was identified. Each RF was individually normalized to remove differences due to average firing rates. The color scale of the RF maps is shown on the scale bar next to the neuron near the bottom right corner.

We estimated the RFs for each neuronal population in V1 by applying a gradient ascent method to optimize corresponding log-likelihood functions with respect to the model parameters to reproduce responses to the set of training visual stimuli (see Materials and Methods). To demonstrate the results of fitting the HSM to a local population of mouse V1 neurons, we plotted the linearized RFs in cortical space with each RF centered on the location of the corresponding neuron’s cell body in the imaged region (Fig 3). The color of the frame around each neuron’s RF represents the value of the non-linearity index (NLI; see Materials and Methods) for the given neuron, indicating the portion of the predicted responses which is due to non-linear as opposed to linear aspects of the HSM, and can be considered as an estimate of a neuron’s non-linearity. We observed a diversity of RF shapes in local regions of mouse V1, with a full range of linear and non-linear characteristics (Fig 3).

### HSM accurately predicts neuronal responses to natural stimuli

**Fig 4:**
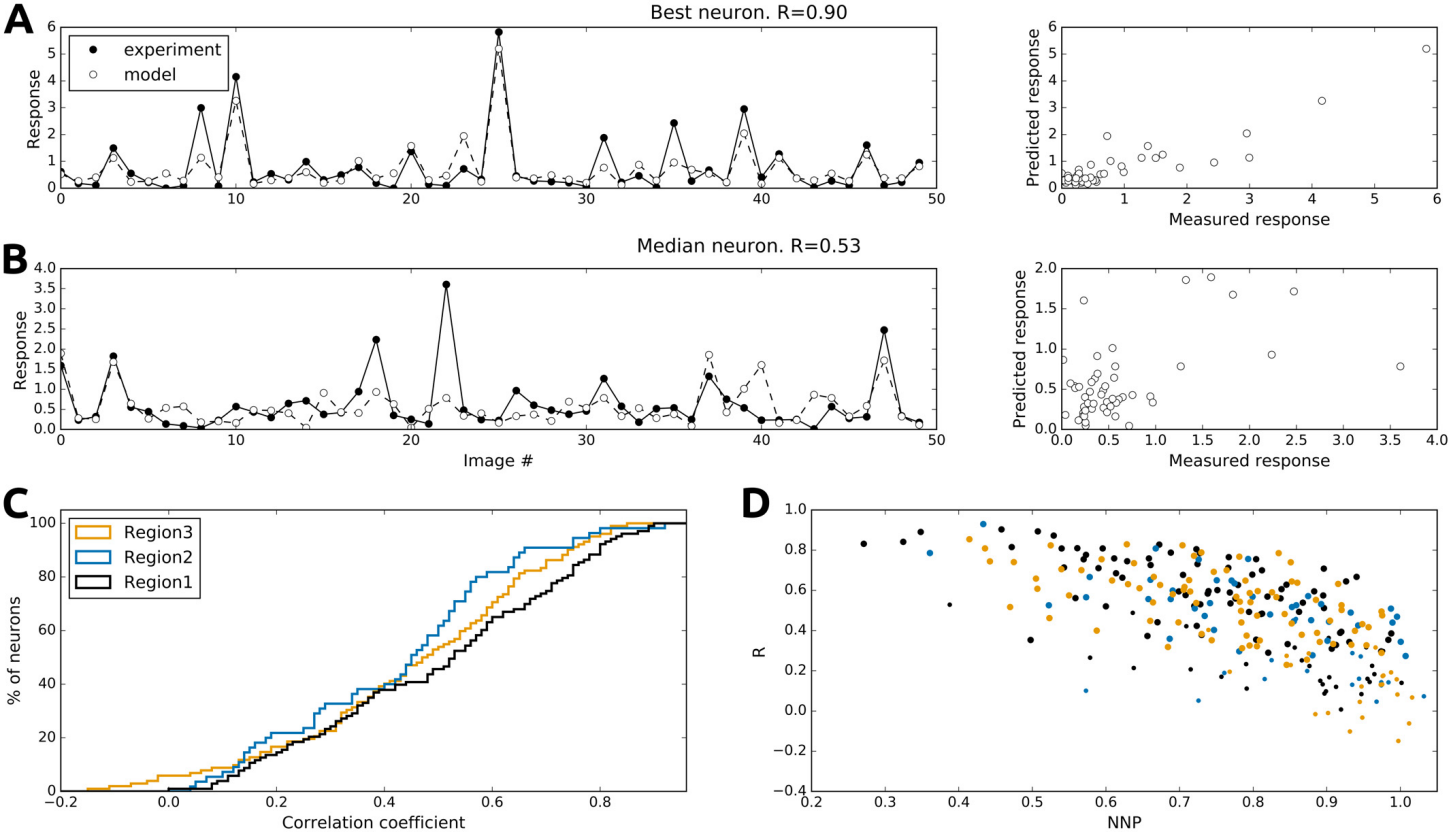
Responses predicted from the HSM are highly correlated with recorded neuronal responses. The measured mean activity (full circles) and the predicted responses (empty circles) for a neuron with highest correlation (R = 0.90, P<0.001)(A) and a neuron with median correlation (R = 0.53, P<0.001)(B). (C) The cumulative distribution functions of correlation coefficients across the population of 260 neurons in the three measured regions. (D) Normalized noise power [28] and the performance of the model are negatively correlated (R = -0.63, -0.55 and -0.67 for the three regions, P<0.001 for all regions). Higher normalized noise power implies lower and more variable prediction performance. The small dots indicate neurons for which the model performance (correlation between predicted and recorded responses) was not statistically significant (based on bootstrapped 95% confidence intervals).

To estimate the performance of the HSM, we measured the correlation between responses predicted by the fitted model and evoked responses to a novel set of validation images (50 images, responses averaged over 8–12 trials) that were not included in the training set (Fig 4). The top neuron depicted in Fig 4A was the best fit neuron (R = 0.9; p<0.001). The model predicted its response with high accuracy, apart from small response deviations at lower response amplitudes. The neuron in Fig 4B exhibited the median correlation between predicted and recorded responses (R = 0.53; p<0.001), where the predicted response captured a considerable part of the neural response, but significant deviations from the measured activity were still observed. The correlation coefficients of neurons from all three imaged regions in V1 were broadly distributed across a range of positive values, with few neurons showing weak anti-correlation (median values of 0.53, 0.45 and 0.47 respectively; p<0.001 for all three regions; Fig 4C). We found a strong negative relationship between the normalized noise power [28] in recordings of individual neurons and the performance of the model for those neurons (Fig 4D). This is because response reliability of neurons has a significant impact on the ability of the model to fit individual neurons, as less reliable responses carry less information about the stimulus, and the mean response from the validation image set is likely to deviate more from the true average response for less reliable neurons. This predicts that collecting a larger training set and increasing the number of repetitions in the validation set would further improve the prediction power of the model.

### HSM outperforms state-of-the-art methods

In the previous section we showed that the HSM can predict well the responses of many neurons to novel natural stimuli. How does the prediction performance of our model compare to other models, including a regularized variant of the linear-nonlinear model (rLN) [9], and the Berkeley wavelet transform (BWT) model [29]? Due to its simplicity and interpretability, the linear-nonlinear model is the standard approach for RF estimation and has been used in a wide range of studies [30]. On the other hand, the recently proposed BWT model (together with the closely related Gabor pyramid variant [18]) represents the state-of-the-art in neural response prediction to naturalistic stimuli, as it can capture linear and non-linear components of the neuronal responses and outperforms most RF identification methods [31].

**Fig 5:**
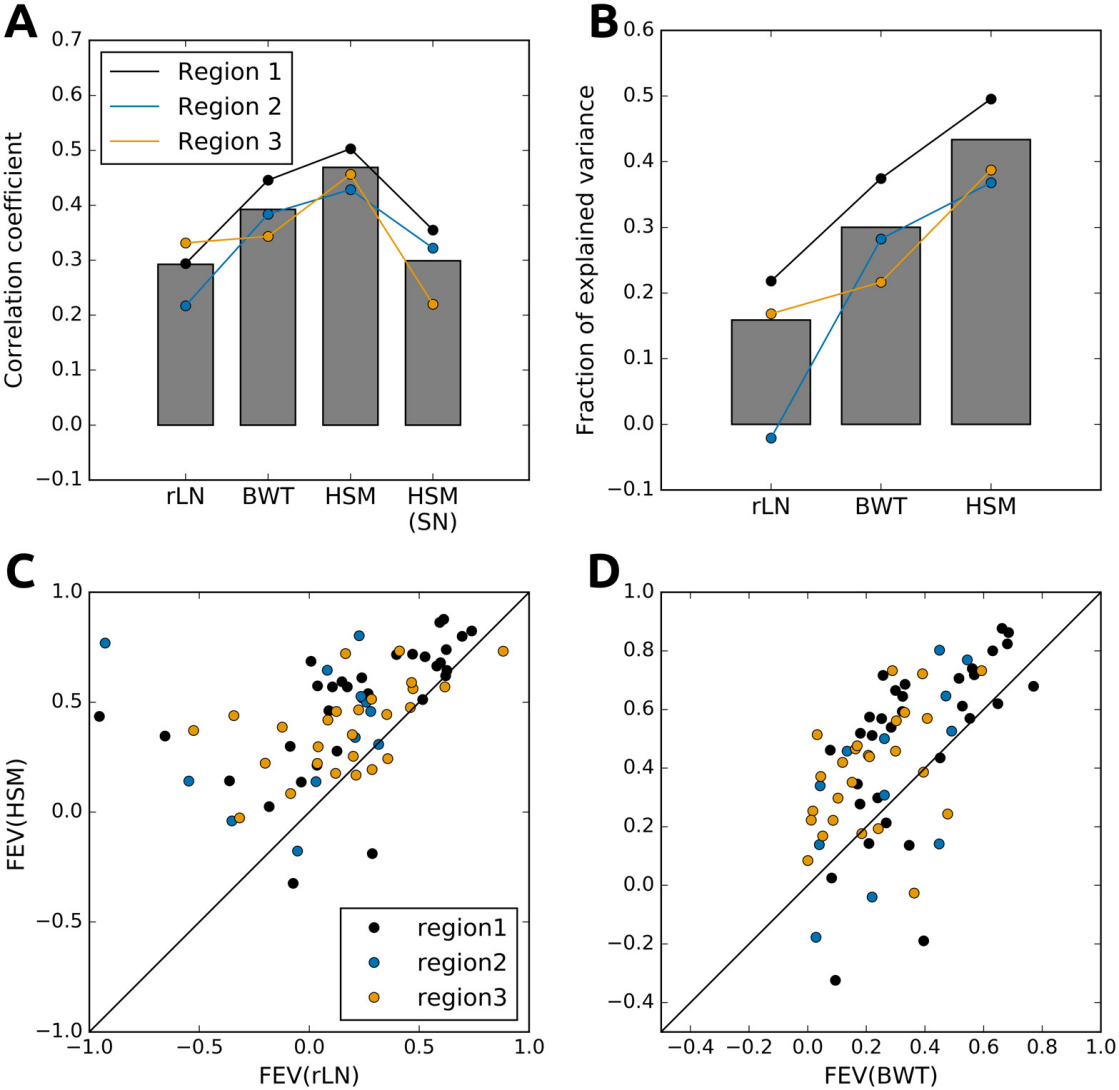
RFs estimated by HSM explain neuronal responses significantly better than existing methods. (A) The average correlation coefficients of the rLN, BWT and HSMs, and of an HSM in which each neuron has been fitted individually HSM(SN). (B) The average fraction of explained variance (FEV) for the three compared models. (C) Scatter plot of the FEV of individual neurons by the rLN and HSMs. (D) Scatter plot of the FEV of individual neurons under the BWT and HSMs. Data from individual regions are marked by the colored lines and/or dots while the averages across regions are indicated as bars.

Fig 5A compares the performance for the three models (measured as the correlation coefficients of measured and predicted responses to a novel set of natural images) averaged across all neurons in the three recorded cortical regions, while the line-graphs show the performance for the three cortical regions separately. The rLN and the BWT methods achieved averaged performances of R = 0.29 and R = 0.39 respectively, while the HSMs outperformed both consistently across all three regions (P<0.001; Wilcoxon signed ranked test; data pooled across the three regions) showing an average correlation of R = 0.47 (a 20% improvement over the BWT model). Furthermore, if we fit the HSM to each neuron individually, we see that the prediction performance drops to the levels shown by rLN (the HSM(SN) condition, R = 0.30). This indicates that the predictive advantage of HSM largely arises from our ability to constrain the fitting problem by the assumption of limited common feed-forward input into a local population of neurons in V1.

To quantify the proportion of the neuronal response captured by our model, we computed the fraction of explained variance (FEV) [28]. Since high quality multi-trial data is required to reliably calculate FEV, for this analysis we excluded neurons with normalized noise power greater than 70% (sparing 70 of the 260 imaged neurons). The average fraction of variance explained by the rLN and the BWT models was 0.16 and 0.30 respectively, while the HSMs outperformed both (P<0.001; Wilcoxon signed ranked test; data pooled across the three regions), achieving an average of 0.43, representing a 43% improvement over the BWT model (Fig 5B). Finally, the improvement in the average prediction (fraction of explained variance) of the HSM was not restricted to subset of neurons, but spread almost across the entire measured population (Fig 5C and 5D).

### Effects of varying LGN input and hidden layer neuron number

The HSM contains two free meta-parameters that are not optimized during the fitting process: the number of LGN units and the number of neurons in the hidden layer (which we express as the fraction γ of recorded neurons in an imaged region). To assess the influence of these parameters on the performance of the HSM, we performed a parameter search. Due to the high computational requirements of the fitting process and the large number of neurons in our dataset we were not able to explore the full space of these two parameters. Instead we performed a partial one-dimensional search through the parameter space, varying one parameter and fixing the other to a value we found empirically to give good performance (9 for the number of LGN units and 20% for the number of hidden units expressed as fraction of imaged neurons). We limited the number of fitting restarts with different initial seeds for each explored value of the meta-parameters to 20.

**Fig 6:**
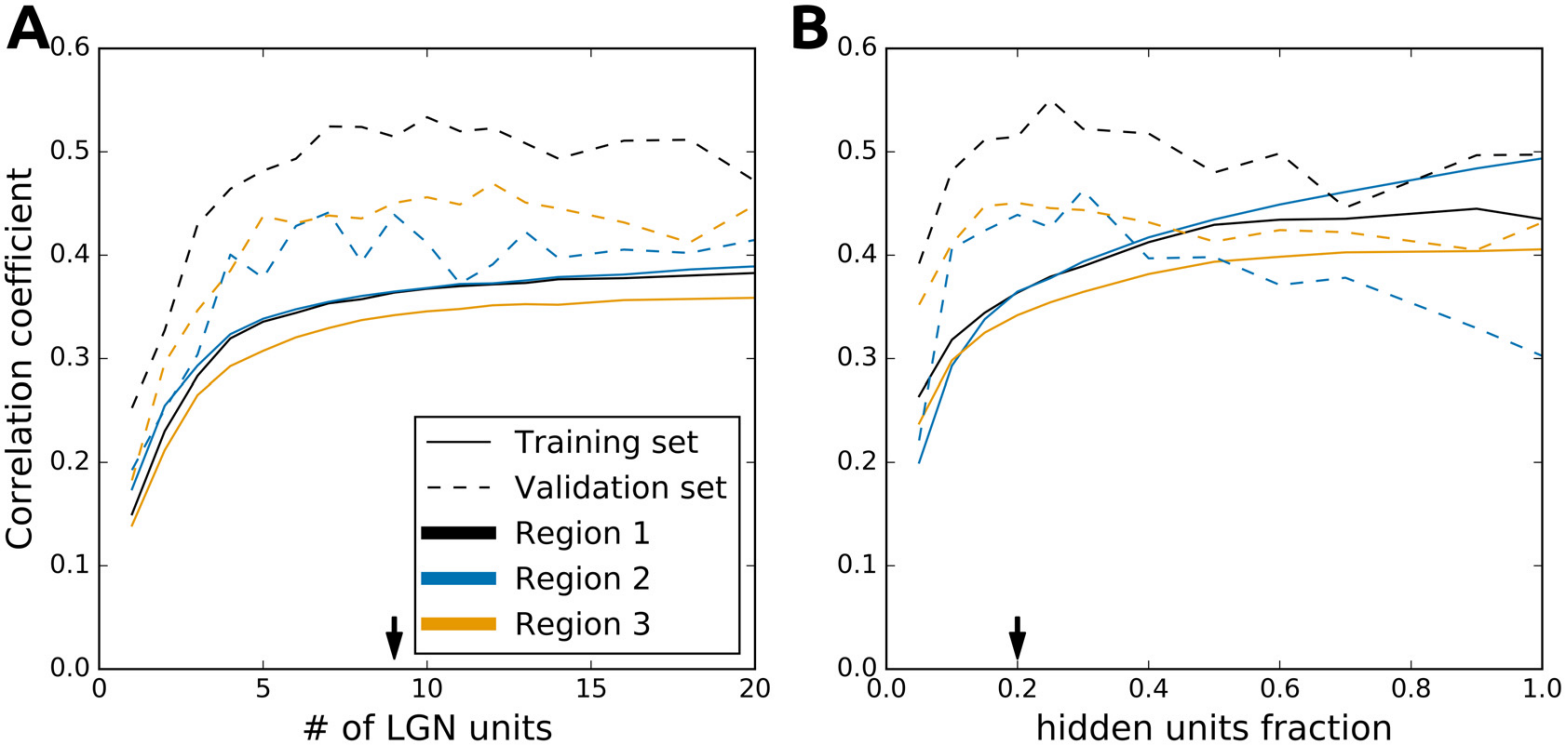
Effect of meta-parameter choice on model performance. (A) Relationship between the performance of the HSM and the number of LGN inputs for the training set (solid line) and the validation set (dashed line). (B) Relationship between the performance of the HSM and the number of units in the hidden layer expressed as fraction of measured neurons for the training set (solid line) and the validation set (dashed line). Note that the performance on the training set is consistently poorer than on the validation set because the validation set is an average over multiple trials while the training set is single-trial data (see Materials and Methods).

The performance of the model on the training set initially increased with the number of LGN inputs (Fig 6A, full lines), however, beyond ~9 LGN units the performance saturated. We observed a similar pattern in the relationship between hidden layer size and performance of the model on the training set (see Fig 6B, full lines). Initially, the performance increased with increasing hidden layer fraction, however, it quickly saturated at a value of ~0.2. A similar relationship between the performance of the model and the meta-parameter values exists when measured against the validation set (Fig 6, dashed lines). Overall, surprisingly few LGN inputs and hidden units are required to capture the responses of large local populations of neurons (>100 neurons). This observation is consistent with the recent evidence showing limited variability in the location of RF subunits in local populations of mouse V1 neurons [19]. It should however be emphasized that the values of these two parameters are very likely an underestimation of the true number of LGN cells innervating the imaged cortical region, and the number of linear (simple) cortical cells from which the imaged neurons receive inputs. These numbers are a reflection of the number of parameters we can resolve given our limited training sets. We expect that larger amounts of data would lead to a slight increase in the number of subunits, and a more accurate correspondence between the fitted model parameters and the underlying neural substrate.

### The nature of the fitted RFs

**Fig 7:**
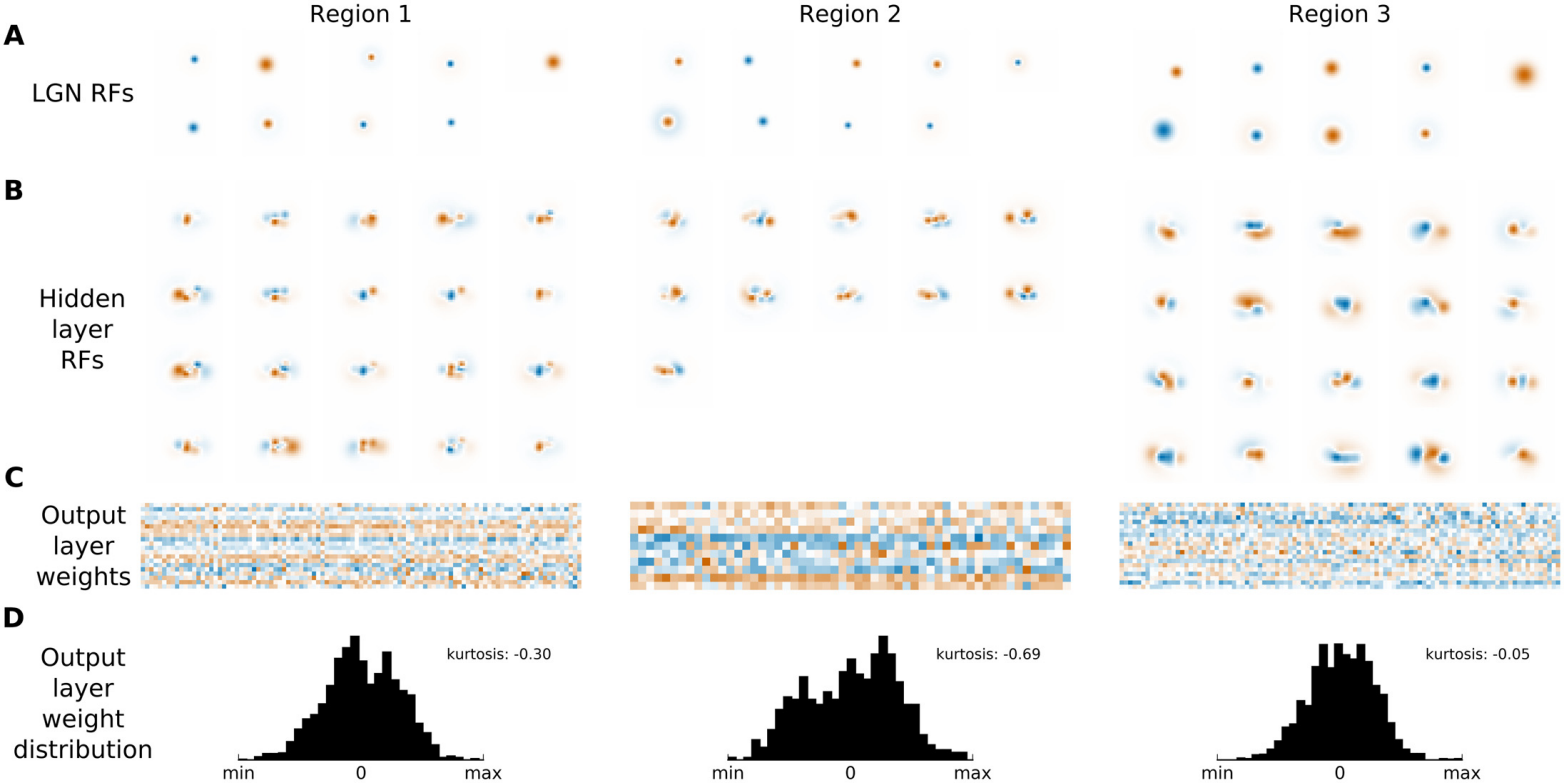
Composition of RFs estimated by the HSM. (A) The kernels of LGN HSM units fitted to the 3 imaged regions in V1. (B) The linear kernels of the hidden units of the HSM fitted to the 3 imaged regions in V1. These have been calculated as the sum of the difference-of-Gaussians kernel of the LGN units weighted by the fitted connections from the LGN to hidden units. (C) The weight matrices between the hidden and output units for HSMs fitted to the 3 imaged regions. For each output neuron the weights were individually normalized. (D) The histograms of the weight matrices shown in C with calculated kurtosis of the respective distributions. Overall, the RFs of the intermediate units in the fitted HSM differ somewhat from standard descriptions of V1 simple cell RFs estimated by rLN (or similar) methods. However, it is possible that the RFs of the hidden units are combined in the HSM output layer to form RFs that—when linearized—match those obtained via the rLN method. To verify this, we performed a rLN analysis using the training set of images and the corresponding responses of the fitted HSM, thus obtaining a linear estimate of the HSM-derived RFs. Fig 8 shows the RFs obtained via the rLN method directly from the data (A columns) and using the corresponding HSM predictions (B columns) for neurons in the first imaged region. Neurons for which a linear RF could be estimated showed a close match between rLN and HSM estimations. This indicates that the fits of the HSM are compatible with the previous rLN results. At the same time, the HSM significantly outperforms response predictions of the rLN model for most neurons, indicating that non-linearities in the HSM, which cannot be captured by the inherently linear rLN model, provide significant performance improvements.

It is important to emphasize that the estimated HSM parameters are unlikely to reflect a direct one-to-one relationship with the underlying biological substrate, but rather offer a functional description of the system. To gain a insight into how the HSM captures the responses of the fitted neurons, we show the linear RFs of all the units in the LGN and intermediate layer of the fitted HSM (Fig 7A and 7B). Note that such linear visualization is not possible for the output layer units, due to the non-linearity of the hidden layer unit transfer functions. Unsurprisingly, as a direct consequence of their definition in the HSM, the LGN kernels have isotropic center-surround structure, but some have very weak surround components. Some intermediate units express RFs that can be well described by Gabor functions, yet others have more unusual shapes, while we also observe multiple cells with similar RFs. This is not surprising given that the number of hidden units used in the HSM models was much smaller (< = 20) than the number of linear (simple) cells that can be expected to reside in the corresponding region of V1. Consequently, it is unlikely that the fitted HSM hidden units correspond to RFs of individual neurons in the imaged area, but rather to a low-dimensional subspace in which neural responses are generated. Advanced model regularization and selection methods could in future improve the link between the HSM units and biological neurons, but these will require collection of substantially more data than available in our experiments to be effective.

**Fig 8:**
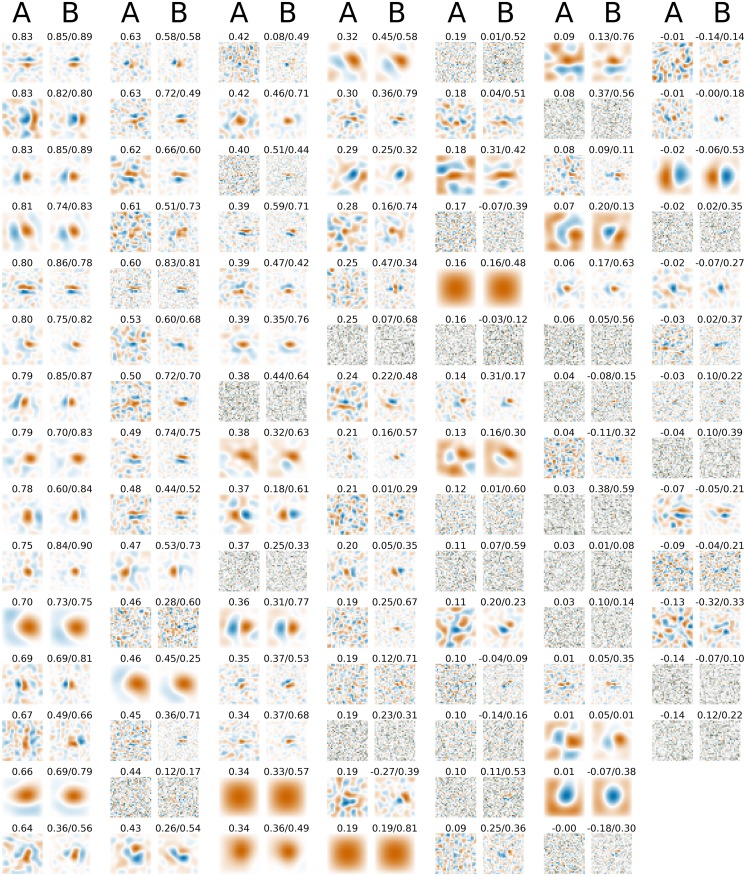
Linear components of RFs estimated with HSM are consistent with rLN-derived RFs. For neurons where RFs can be discerned from the noise, the rLN model produces very similar RFs for both experimental responses (A columns) and HSM responses (B columns), indicating that the HSM is compatible with the RFs estimated by rLN. The numbers above the rLN filters obtained directly from data (A columns) show the performance (R) of the rLN model for the corresponding neuron. The two numbers above the rLN filters obtained using the prediction of the HSM (B columns) indicate the performance of the rLN model derived from the HSM prediction (before slash) and the performance of the full HSM for the corresponding neuron (after slash).

How then are the hidden layer RFs combined to generate the fitted RFs of the measured neurons in the HSM? Fig 7C shows the weight matrices between the hidden and output layer for all three regions. Each weight matrix is relatively dense. Based on theories about complex cell construction including the energy model [32] or the STC analysis, which suggest a relatively limited number of linear filters in complex cells [7,10], one would expect these matrices to be sparse. This does not appear to be the case in the HSM, as is suggested by the negative kurtosis values of the weight distributions Fig 7D. One possibility is that these dense weight matrices are the consequence of over-fitting. Another option is that the model hidden units correspond to linear presynaptic cells (or linear combinations of a set of such cells) and the dense weight matrices between the hidden and output layer reflect the fact that single cortical neurons receive a large number of connections from other local cortical neurons [33]. Finally, the small number of hidden HSM units means that a combination of larger numbers of them might be needed to obtain the RFs of the individual recorded neurons, leading to a lack of sparseness in the weight matrices. In the future, simultaneous imaging of layer 4 and 2/3 will be required to resolve this question experimentally.

**Fig 9:**
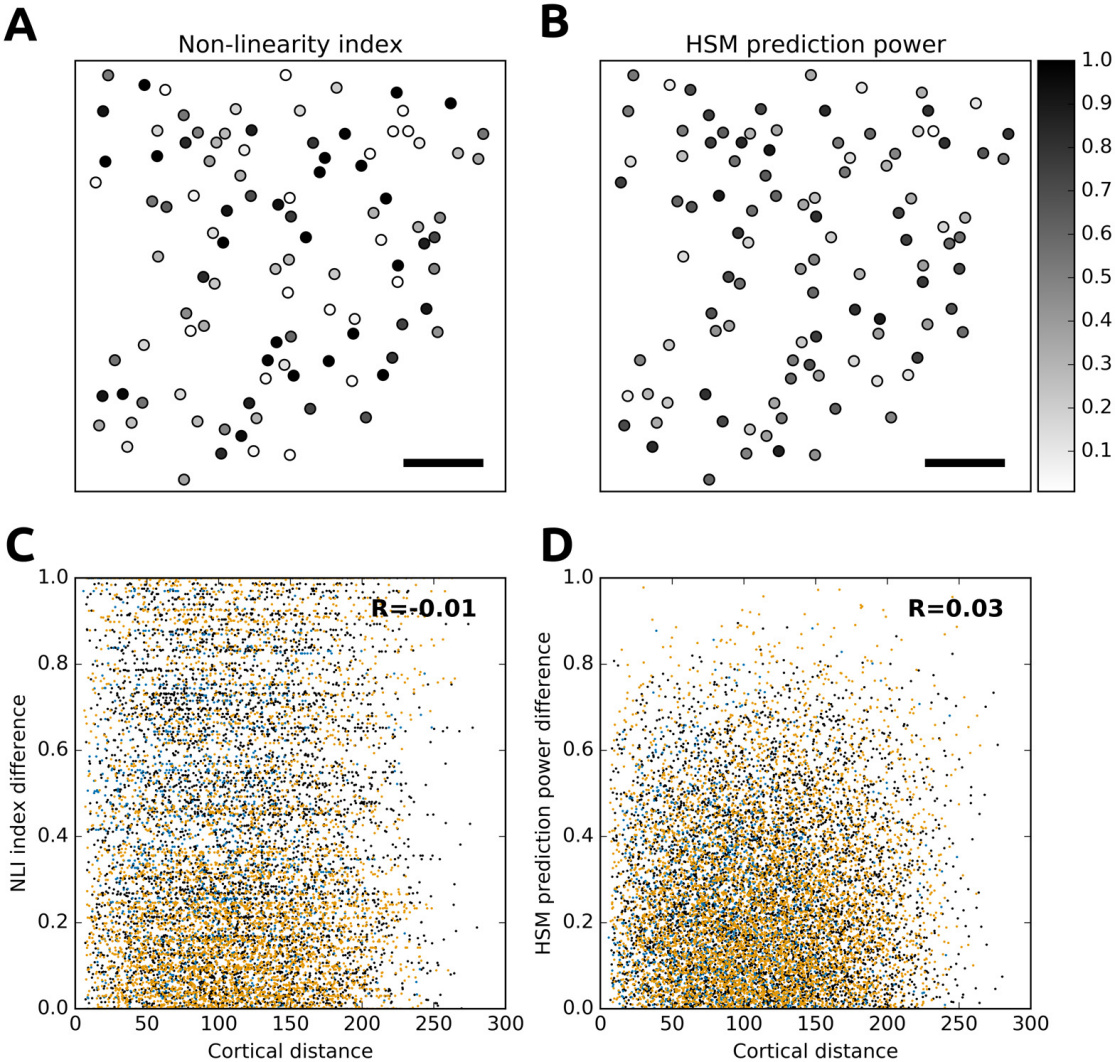
Distribution of NLI index and HSM prediction power in cortical space. (A-B): The scatter plots of the NLI index and HSM prediction power in cortical space from an example cortical region. The points correspond to the positions of cell bodies in cortical coordinates and the gray levels correspond to the two measures (first region shown, scale bar 50μm). (C-D) No correlation between cortical distance and NLI and HSM prediction power. The three recorded cortical regions correspond to the black, blue and orange colors. For both measures and all regions, all correlation coefficients were small (R<0.05) and their signs were not consistent across regions.

Finally, we examined the evidence for spatial organization of two model measures in the local cortical network (Fig 9). We found no evidence for a relationship between cortical distance and non-linearity index (Fig 9A and 9C; R < 0.05, P>0.1) or model prediction power (Fig 9B and 9D; R < 0.05, P>0.1). These results are consistent with the findings of several previous studies that—with the exception of retinotopic position—failed to find a spatially organized arrangement of neurons of similar RF properties in local cortical networks of rodent V1 [21,34].

## Discussion

In this study we introduce a novel model-based method for estimating RFs simultaneously in large populations of V1 neurons, under the assumption that the population shares input from a limited number of thalamo-cortical afferents. We applied the novel model (HSM) to recordings of local populations of neurons in L2/3 of mouse V1 in response to natural images. Our model can explain significant proportions of signal variance of reliably responding neurons in V1 and improves upon existing rLN [11] and BWT models [29] when applied to the same data.

The improved performance of HSM in comparison to the rLN is due to its greater expressive power. The two layers of non-linearity allow the HSM to account for responses of non-linear cells, such as the complex cells in V1. Furthermore, we have also tested an advanced linear method, the automatic locality determination (ALD) [35], but when applied to our data this method showed very similar performance to the rLN model, yielding correlations in the validation set of 0.31, 0.24 and 0.299 in the three recorded regions. When compared to the BWT model, HSM still has the advantage in expressiveness for it allows constructions of RFs that deviate from the stereotypical Gabor-like RFs imposed into BWT structure. Moreover, HSM better constrains the optimization problem by assuming shared afferent input into a cortical column. Thus, unlike rLN or BWT, HSM fits all recorded neurons simultaneously. This makes the HSM parameters constrained by the responses of all recorded neurons, effectively increasing the amount of data available for fitting. Finally, it should be noted that we have also attempted to apply the STC method to the presented data. However, due to the dependence of STC on very large datasets, when applied to our limited data, STC failed to identify any significant eigenvectors for the majority of neurons [10]. This further highlights the advantages of the HSM in estimation of non-linear RFs from limited data.

Recent experimental studies have indicated that local populations of V1 neurons in cats and mice share a limited number of inputs from LGN [19] [25]. Here we offer further support for this hypothesis, by showing that a model assuming feed-forward convergence of thalamic afferents, shared among a population of neighboring cortical neurons, resulted in RF estimates with better predictive power than previous models (see Fig 5). Additionally, removing the assumption of pooled hierarchical input resulted in dramatic drop in quality of estimated RFs (see Fig 5). We show that as few as 9 LGN-like units are sufficient to explain a significant proportion of the stimulus dependent responses in a local population of L2/3 neurons within a ~300x300μm field of view. This is particularly remarkable given the diversity of RFs observed in local populations of mouse V1 neurons (Fig 3) [34].

It is important to emphasize that the estimated structure of the HSM cannot be interpreted as direct evidence of the underlying connectivity. This is obvious in the case of the hidden model units which are orders of magnitude fewer in the HSM models used in this study than the expected number of layer 4 neurons within the corresponding region of mouse V1. It is also very likely that we underestimate the actual number of thalamic neurons innervating the imaged regions of mouse V1. This might be because multiple LGN neurons with similar RFs will be approximated with a single LGN DoG model, consistent with a recent study that directly mapped the RFs of LGN axons in V1 [36]. Further advances in recording techniques that will allow collection of more data (both in terms of image presentation and sampling ratio of the local neural population) should impose more constraints on the fitting of HSM parameters and thus offer a closer picture of the underlying neural substrate. Furthermore, additional prior knowledge (if available), such as the cortical depth/layer membership of the neurons, or neural type, could be incorporated into the HSM model to further constrain its parameter estimation.

Unlike most previous approaches to RF estimation, the optimization problem HSM poses is not convex, and thus finding the global optimum is not guaranteed. But HSM still outperforms other methods because the non-convexity of the optimization is favorably compensated by its better expressive power. Importantly, the quality of the RF estimation is determined by the optimization algorithm used to fit the HSM parameters. We found the truncated Newton conjugate method worked well for the present form of the model, but adding further nonlinearities into the model dramatically decreased its performance. This is likely because adding nonlinearities transformed HSM into a so-called “deep learning” problem which is known to be difficult to optimize. However, the recent advances in optimization techniques applicable to deep-learning problems [37] could improve the fitting of HSM and allow inclusion of additional nonlinear mechanisms.

The limited number of natural images that we could present during each imaging experiment and the relatively slow time-course of spike-related calcium signals constrained this study in three important ways. First, we did not consider the temporal properties of RFs, because the slow sampling (7.6Hz) and kinetics (100s milliseconds) of the calcium signals did not lend this dataset to the analysis of fine-scale temporal response properties of RFs. Second, we did not utilize ‘early-stopping’ criteria to prevent over-fitting, as they require an extra dataset to be separated out of—in our case very limited—training set. We found that such reduction of the training set outweighed the gains due to early stopping. Third, estimation of couplings between neurons using the GLM method has previously been shown to greatly improve prediction power. However, we did not include coupling filters in HSM, as their estimation relies on fine-scale temporal sampling of the recorded neural activity [2]. Improvements in functional imaging, including higher sampling rates, improved signal-to-noise ratio, faster calcium indicator kinetics, voltage-based indicators, and better spike estimation techniques, will allow for more accurate reconstructions of underlying spike-trains in large populations of imaged neurons. Moreover, recent applications of genetically encoded calcium indicators and chronic preparations [38,39] could greatly increase the number of possible stimulus presentations in anaesthetized and awake mice. Overall, such advances would make it possible to overcome all the limitations discussed above, and thus provide a framework for further improvements in understanding the relationship between connectivity and functional properties of V1 and higher visual cortical areas.

## Materials and Methods

### Animals and surgical procedures

All experimental procedures were carried out in accordance with institutional animal welfare guidelines and licensed by the UK Home Office. Experiments were performed on C57Bl/6 mice between postnatal day 30 and 40. Mice were anesthetized with a mixture of fentanyl (0.05 mg/kg), midazolam (5.0 mg/kg), and medetomidine (0.5 mg/kg). During Ca^2+^-imaging experiments, light anesthesia was maintained by Isoflurane (0.3–0.5%) in a 60:40% mixture of O_2_:N_2_O delivered via a small nose cone. Surgically, a small craniotomy (1–2 mm) was carried out over primary visual cortex and sealed after dye injection with 1.6% agarose in HEPES-buffered artificial cerebrospinal fluid (ACSF) and a cover slip.

### Dye-loading and Ca^2+^-imaging

For bulk loading of cortical neurons the calcium-sensitive dye Oregon Green Bapta-1 AM (OGB-1 AM; Molecular Probes) was first dissolved in 4 μl DMSO containing 20% Pluronic, and further diluted (1/11) in dye buffer (150 mM NaCl, 2.5 mM KCl and 10 mM HEPES (pH 7.4)) to yield a final concentration of 0.9 mM. Sulforhodamine-101 (50 μM, Molecular Probes) was added to the solution for experiments in C57Bl/6 mice to distinguish neurons and astrocytes [40]. The dye was slowly pressure injected into the right visual cortex at a depth of 150–200 μm with a micropipette (3–5 MΩ, 3–10 psi, 2–4 min) under visual control by two-photon imaging (10x water immersion objective, Olympus). Activity of cortical neurons was monitored by imaging fluorescence changes with a custom-built microscope and a mode-locked Ti:sapphire laser (Mai Tai, Spectra-Physics) at 830 nm through a 40x water immersion objective (0.8 NA, Olympus). Scanning and image acquisition were implemented in custom software (Labview, NI). The average laser power delivered to the brain was <50 mW.

### Data acquisition in Ca^2+^-imaging experiments

Imaging frames of 256x256 pixels were acquired at 7.6 Hz. After each recording the focal plane and imaging position was checked and realigned with the initial image if necessary. Image sequences were aligned for tangential drift and analyzed with custom programs written in ImageJ (NIH), Matlab (Mathworks) and Labview (NI). Recordings with significant brain movements, vertical drift, or both were excluded from further analysis. Cell outlines were detected using a semi-automated algorithm based on morphological measurements of cell intensity, size, and shape, and subsequently confirmed by visual inspection. After erosion of the cell-based regions of interest, to minimize influence of the neuropil signal around the cell bodies, all pixels within each region of interest were averaged to give a single time course (ΔF/F), which was additionally high-pass filtered at a cut-off frequency of 0.02 Hz to remove slow fluctuations in the signal. Unresponsive neurons during spontaneous and evoked conditions were excluded from further analysis, by testing whether, for each cell, the distribution of all fluorescence values was not significantly different (i.e. positively long-tailed) from a random, normal distribution (Kolmogorov-Smirnov goodness-of-fit test). Astrocytes labeled with Sulforhodamine 101 (red fluorescence) were excluded from the analysis.

Spike trains were inferred from calcium signals using a fast non-negative de-convolution method which approximates the most likely spike train for each neuron, given the observed fluorescence [22]. The deconvolved traces represent estimates proportional to the number of action potentials emitted during the corresponding period. These proportional estimates were calibrated based on simultaneous cell attached recordings and calcium imaging in individual neurons [23] to represent the estimated number of emitted spikes. The inferred spike trains were further processed by computing the sliding average with a window of three frames. This was done to offset the biases introduced by the temporal quantization due to the relatively slow data acquisition rate.

### Stimulus presentation protocol and data pre-processing

Stimuli were presented on 60 Hz LCD monitors, at a resolution of 1024×768 pixels. A retinotopic mapping protocol was used to ensure that the monitor covered the RF of recorded neurons: a patch of moving gratings was presented at 12 different locations on the screen, for 1.4 s in each location with a gap of 1.5 s between locations. The monitor was repositioned such that the preferred retinotopic position of most imaged neurons was roughly in the middle of the monitor.

The stimulus set was composed of static scenes from David Attenborough’s BBC documentary Life of Mammals, depicting natural scenes such as landscapes, animals or humans. Images were scaled to have 256 equally spaced luminance steps, and were composed of 384×208 pixels, and expanded to fill the screen. Each image appeared in the stimulus set four times, in the original form, flipped horizontally and flipped vertically, and with reversed contrast. The onset of image presentation was aligned with the frame rate of the scanning.

To account for the noise and the dynamics of somatic calcium signals, we applied a relatively slow visual stimulation protocol (total time = 1974 ms per image presentation, i.e. 15 imaging frames at 7.6 Hz) to obtain reliable responses of V1 neuronal populations to the naturalistic stimuli. Images were presented for 500 ms, and interleaved with blank grey screens presented for 1474 ms (see Fig 1A). Averaging of the resulting calcium traces across all stimulus presentations and neurons revealed the typical onset and offset dynamics of the neural responses. We define the response of a neuron to single image presentation as the average number of spikes inferred by the spike extraction algorithm across imaging frames 3–7, which we identified to hold the bulk of the onset signal, but likely also an early component of the offset response. This way, for each imaged region, we obtained two datasets of values. The first is an *n*×*m* matrix corresponding to the responses of each of the *m* recorded neurons to *n* single trial image presentations, which we refer to as the training set (see Fig 1B). Additionally, in each region we recorded responses to 8–12 presentations of another 50 images forming the second dataset, a 50×*m*×*r* matrix, we will refer to as the validation set. Three regions in two animals were recorded, containing 103, 55 and 102 neurons, while presenting sequences of 1800, 1260 and 1800 single trial images, respectively. The images were presented in partially interleaved manner. The training images were divided into 10 blocks. Additional blocks were formed by the 50 validation images, in each of these blocks the 50 images were presented multiple times. During the experiment the resulting stimulation blocks were presented in random order.

For each region, we ran a rLN fitting protocol with full-field stimuli to determine the rough position and size of all the neurons' RFs. Consistent with retinotopic map in mouse V1, in all three recorded regions all recovered RFs were located in a restricted region of visual space. This allowed us to determine a region of interest in the visual space, centered on the set of initially recovered RFs and spanning roughly two times the area they covered. The images were constrained to this region of interest and then down-sampled to 31×31 pixels to form the input stimuli set, which was used in all the subsequent analysis.

### HSM

The HSM is a feed-forward network consisting of three fully connected layers (see Fig 2). The first layer corresponds to units found in LGN, represented as difference-of-Gaussian kernels. The output of the *i*-th unit in the LGN layer to an image *I* is computed as follows:

$$\psi_{i1}\;=\;\underset{k,l}{\sum }I_{kl}\left(\frac{\alpha_{i}}{\sigma_{i}^{2}}\;e^{-\frac{{\left(k-\mu_{i}^{x}\right)}^{2}+\;{\left(l-\mu_{i}^{y}\right)}^{2}}{2\sigma_{i}^{2}}}-\frac{\beta_{i}}{\rho_{i}^{2}}\;e^{-\frac{{\left(k-\mu_{i}^{x}\right)}^{2}+\;{\left(l-\mu_{i}^{y}\right)}^{2}}{2\rho_{i}^{2}}}\right)$$ (1)

where *ψ*_*i*1_ is the output of the *i*-th unit in the first layer containing LGN units, *I*_*kl*_ is the image intensity at coordinates *k* and *l*, *σ*_*i*_ and *ρ*_*i*_ are the widths of the center and surround Gaussians of the *i-*th LGN unit, $\mu_{i}^{x}$ and $\mu_{i}^{y}$ are the *x* and *y* center coordinates of the *i*-th LGN unit, and *α*_*i*_ and *β*_*i*_ are the weights of the center and surround respectively.

The two ‘cortical’ layers consist of simple linear integrators with a logistic-loss output non-linearity:

$$\psi_{il}=\;f\left(\underset{j}{\sum }w_{ij}\psi_{j\left(l-1\right)}\right)$$ (2)

where *ψ*_*il*_, *l* ∈ {2,3}is the output of the *i-*th unit in layer *l*, *w*_*ij*_ is the weight from unit *j* to unit *i* and *f* is a logistic-loss transfer function:

$$\text{f}\left(\text{x}\right)\;=\text{ log}\left(1+\text{exp}\left(\text{x}-{\text{t}}_{\text{i}}\right)\right)$$ (3)

where *t*_*i*_ is the threshold of unit *i*. Thus, the free parameters of the model are the 6 parameters per LGN unit (*α*_*i*_,*β*_*i*_,$\mu_{i}^{y},\mu_{i}^{y},\;\sigma_{i}$,*ρ*_*i*_), one parameter per each cortical unit corresponding to its threshold *t*_*i*_ and the weights between the layers, totaling 6*s*_1_ + *s*_2_ + *s*_3_ +*s*_1_*s*_2_ + *s*_2_*s*_3_ parameters, where *S*_*i*_ is the size of the layer *i*. Assuming Poisson spiking, we fit the HSM via a maximum-likelihood method by performing gradient descent on the corresponding log-likelihood function [41]:

$$\text{log }p\left(y|x,\phi \right)=\;\underset{i}{\sum }y_{i}\text{log}M\left(\phi ,x_{i}\right)-\underset{i}{\sum }M\left(\phi ,x_{i}\right)$$ (4)

where *y* are the measured neural responses, *x* are the input patterns, *ϕ* are the free parameters of the model and $\;M\left(\phi ,x_{i}\right)={\vec{\psi }}_{3}$ is the output of the model to image *x*_*i*_. To implement the model and search for optima of its log-likelihood function we use the theano package [42,43] in combination with the constrained truncated Newton conjugate method (implemented by the Python scipy.fmin.tnc function). This optimization method allows constraining the parameters to lie within intervals, allowing us to enforce the centers of LGN units to lie within the image, and the width of the center and surround Gaussians of LGN units to be positive and smaller than the width of the image.

The model has two free meta-parameters that are not set by the fitting process: the number of LGN units (*s*_1_) and the number of units in the hidden layer (*s*_2_ = *γs*_3_), where *γ* expresses the number of hidden units as a fraction of the number of output units. We performed a systematic search for these two parameters with respect to the performance of the model on the training set. We found that the performance of the model on the training set quickly saturates when increasing the number of LGN inputs and the fraction of hidden units, at values of about *s*_1_ = 9 and *γ* = 0.2 respectively. Therefore, in order to prevent over-fitting and facilitate simple comparison, we decided to use these low values of the two free meta-parameters for fitting of all the three regions, in all the other analysis.

The optimization problem posed by the HSM log-likelihood function is not convex. Therefore we are not guaranteed to find a global optimum and thus the solution found, and consequently its performance, will be dependent on the initial parameter values. To lessen this dependence on initial parameters, each fitting was run multiple times with different randomly seeded initial parameter values, and followed by selecting the model with the best performance on the training set. The initial model parameterizations were obtained by specifying ranges for each HSM parameter, and then randomly selecting values uniformly from within these ranges. For example for the position parameters of the LGN DoG kernels we set the ranges to correspond to the extent of the input images. For the parameter search experiments we performed 20 restarts of the fitting algorithm for each meta-parameter combination, while for the rest of the analysis with the selected meta-parameters we performed 50 restarts.

To show that the initial parameter restarts are an effective method, we have fitted the HSM model using 100 different initial parameterizations (S1 Fig). The performance of the fitted HSM model on the training set was correlated with the performance on the validation set across the set of initial seeds (panel A in S1 Fig). Even though the different initial conditions lead to solutions with similar responses and prediction power (S1 Fig), these solutions correspond to considerably different estimates of the HSM parameters (even if we account for some basic ambiguities: see S1 Fig caption for details). We observe analogous results if we fix the initial conditions, but instead fit HSM on 100 sub-samples of the training set (S2 Fig). Overall, the HSM method is successful in consistently finding good functional descriptions of a neuron’s stimulus-response function, but these solutions are not unique, and many different HSM parameterizations can lead to the same input-output relationships. Such many-to-one mappings between HSM parameterizations and input-output functions are consistent with previous observations in machine learning and computational neuroscience [44–47]. It remains to be seen if incorporating additional constraints by increasing the amount of data for training, or obtaining more complete samples of local neuronal populations, or obtaining other experimental observables (e.g. layer membership or cell type of the recorded neurons) or further extending the HSM model (eg. with coupling filters) could lead to HSM parameterization that is less sensitive to initial conditions and more closely reflects the underlying biological substrate.

The exact time the model training took varied based on the free model parameters, the number of output neurons, and the size of the training set. For the first region (103 neurons, 1800 training examples) and the values of the free parameters used in the model comparison (see Fig 5) the fitting (a single initial condition) took approximately 3 hours on a modern 2.4MHz CPU with 4GB of memory.

To allow for visualization and comparison of the fitted model and to facilitate further analysis we also performed linearization of the model. We did this by fitting the rLN model to the HSM responses to the training set of images. Furthermore, in order to quantify the extent to which the accounted-for portion of the neuronal response predicted by the HSM is linear or non-linear, we define a non-linearity index (NLI) as:

$$NLI\;=\frac{LC\;-\;\text{max}\left(LLC,0\right)}{LC}$$ (5)

where *LC* corresponds for the correlation between the activities predicted by the HSM and measured activities in response to the validation set of images, and LLC is the corresponding value but for the linearized HSM. For 53 of the 259 neurons for which *LC* < *LLC* we set *NLI* to zero. For a given neuron, *NLI* will be zero if the correlation between measured activities and predicted activities by HSM is entirely accounted for by the linearized model alone, while the value is 1 if the linear model does not account for any of the correlation—and thus the correlations are due to non-linear aspects of the HSM.

### rLN model and the wavelet decomposition model

In order to assess the performance of the HSM against other RF decoding methods we fitted the same data with a regularized least-squares variant of the LN model [11], as well as with a recent method using nonlinear Berkeley wavelet transform (BWT) decomposition of the stimuli [8,29].

#### rLN

We used a variant of the linear-nonlinear model with Laplacian regularization [11] that has been shown to offer superior results over basic LN models, when applied to datasets composed of naturalistic stimuli. Following the filter estimation, we have also fitted the point non-linearity which significantly increased the performance of the rLN estimates. Briefly, the method is similar to ridge-regression estimation; however, instead of imposing minimal values on individual pixels it imposes a minimal Laplacian in each pixel of the estimated kernel, thus directly biasing the estimated kernels to be locally smooth:

$$\hat{k}=\;pinv\left(S^{T}\;S\;+\;\alpha L\right)S^{T}\;r$$ (6)

where *k* is the resulting kernel estimate, *S* is the stimulus matrix, *L* is the regularization matrix expressing the Laplacian constraints, *α* is the regularization constant determining the strength of the regularization, *r* is the measured responses of the neuron to the stimuli in *S*, and *pinv* is the Moore-Penrose pseudoinverse of a matrix. We selected the value of the meta-parameter *α* by measuring the performance of the model with respect to different values of the *α* parameter on a portion (10%) of the training set set aside for testing. This yielded a smooth function with a clear maximum. The value of the *α* parameter at this maximum was used in all the following performance comparisons. Next, after obtaining the estimate of the linear kernel *k* we used the training set to estimate the shape of the point non-linearity by doing a simple histogram fit at 20 regular intervals. To predict a response of a neuron given the output *r* of the estimated linear kernel, we find the two bins closest to *r* and use a linear interpolation between these two with respect to *r* to estimate the response of the neuron. Increasing or decreasing the number of bins slightly did not change the performance significantly. Overall, we found that both Laplacian regularization and the point non-linearity estimation greatly improved the performance of the model in comparison to basic rLN or the ridge-regression variant.

#### Wavelet decomposition model

A detailed description of the wavelet decomposition model can be found in [8,29]. Briefly, the model consists of two stages. First the stimuli are decomposed using a bank of wavelet filters [29], and the response of each filter is half-rectified taking the positive and negative responses separately, forming a vector of half-rectified wavelet responses for each input. The second stage corresponds to a linear model where the final response of the neuron is described as the weighted sum of the half-rectified wavelet responses. This linear model is fitted by minimizing the standard mean-squares error loss function, using the L2Boost algorithm [48]. This algorithm iteratively adjusts only a single weight along the steepest gradient. Combined with early stopping to prevent over-fitting, this procedure tends to provide a sparse representation of the linear model.

We used the implementation of the above wavelet decomposition method provided by the freely available STRFLab package [49]. Due to the fact that our stimulation protocol suppressed the temporal properties of the neuron’s RF, and because single points in our data set represent the average activity over several hundred milliseconds, we set the temporal size and the number of velocities in the BWT to one, and the list of delays at which the RF was fitted was set to zero. Furthermore, the number of phases and the number of orientations in the BWT was set to 4 and 12 respectively and 2D Gaussians were also included in the set of pre-processing filters. We experimented with several values of these meta-parameters, and picked those showing best performance, to ensure that the BWT model is not disadvantaged in this comparison. The prediction power of both the rLN and Wavelet decomposition model was evaluated on the same separate validation data set as the HSM.

## Supporting Information

S1 Dataset Dataset of stimuli and corresponding population responses of recorded neurons. The dataset contains the natural image stimuli used in this study, and the pre-processed population responses of mouse V1 neurons recorded in the 3 cortical regions analyzed in this study.
(ZIP)

S1 Model Python implementation of the HSM model. (ZIP)

S1 Fig Analysis of sensitivity of HSM to starting conditions. (A) The model performance on the training vs. validation data set across 100 HSM fits using different initial conditions. The three plots show results for each of the 3 imaged regions separately. The color coding of the regions is the same as throughout the main paper. (B) The correlations between responses of pairs of HSM model fits obtained from different initial conditions. (C) The RFs of matched LGN units of three different fits of the HSM model using different initial conditions (the selected initial conditions are marked in A as seed A,B and C). (D) Matched hidden unit RFs. The ordering of the LGN and hidden units in the HSM is arbitrary which complicates comparison of fitted parameters from different initial conditions. When comparing two model parameterizations, in an ideal case, we would like to find a permutation of the LGN and hidden units that maximizes the similarity (for example measured as the mean correlation across corresponding units) between the two sets of units. Finding such permutation is however intractable. Here we have employed simple greedy strategy to match the two sets of units. In C and D the units from seed B were matched to units from seed A and independently the units from seed C were matched to units from seed A. Furthermore, there is redundancy in the HSM model between the polarity of the LGN units and the weights from the LGN units to hidden units, which are not required to be only positive. For this reason the matching of the LGN units is based on the absolute values of their correlations, and for the visualization the LGN units are flipped such that their polarity matches. (E) The weights from hidden to output units.
(TIF)

S2 Fig Analysis of sensitivity of HSM to different re-samplings of training set. (A) The model performance on training vs. validation data set across 100 HSM fits using different sub-samples of the training set. Each sample was obtained by removing 100 random training stimuli. The three plots show results for each of the 3 imaged regions separately. The color coding of the regions is the same as throughout the main paper. (B) The correlations between responses of pairs of HSM model fits obtained from different training set samples. (C) The RFs of matched LGN units of three fits of HSM to three different samples of training set (the selected samples are marked in A as seed A,B and C). (D) Matched hidden unit RFs. See panel D of S1 Fig caption for details about the matching procedure. (E) The weights from hidden to output units.
(TIF)
