# Supplementary figures and images for: Model Constrained by Visual Hierarchy Improves Prediction of Neural Responses to Natural Scenes
Source: PLoS Comput Biol. 2016 Jun 27;12(6):e1004927. doi: 10.1371/journal.pcbi.1004927 (PMC4922657; doi:10.1371/journal.pcbi.1004927)

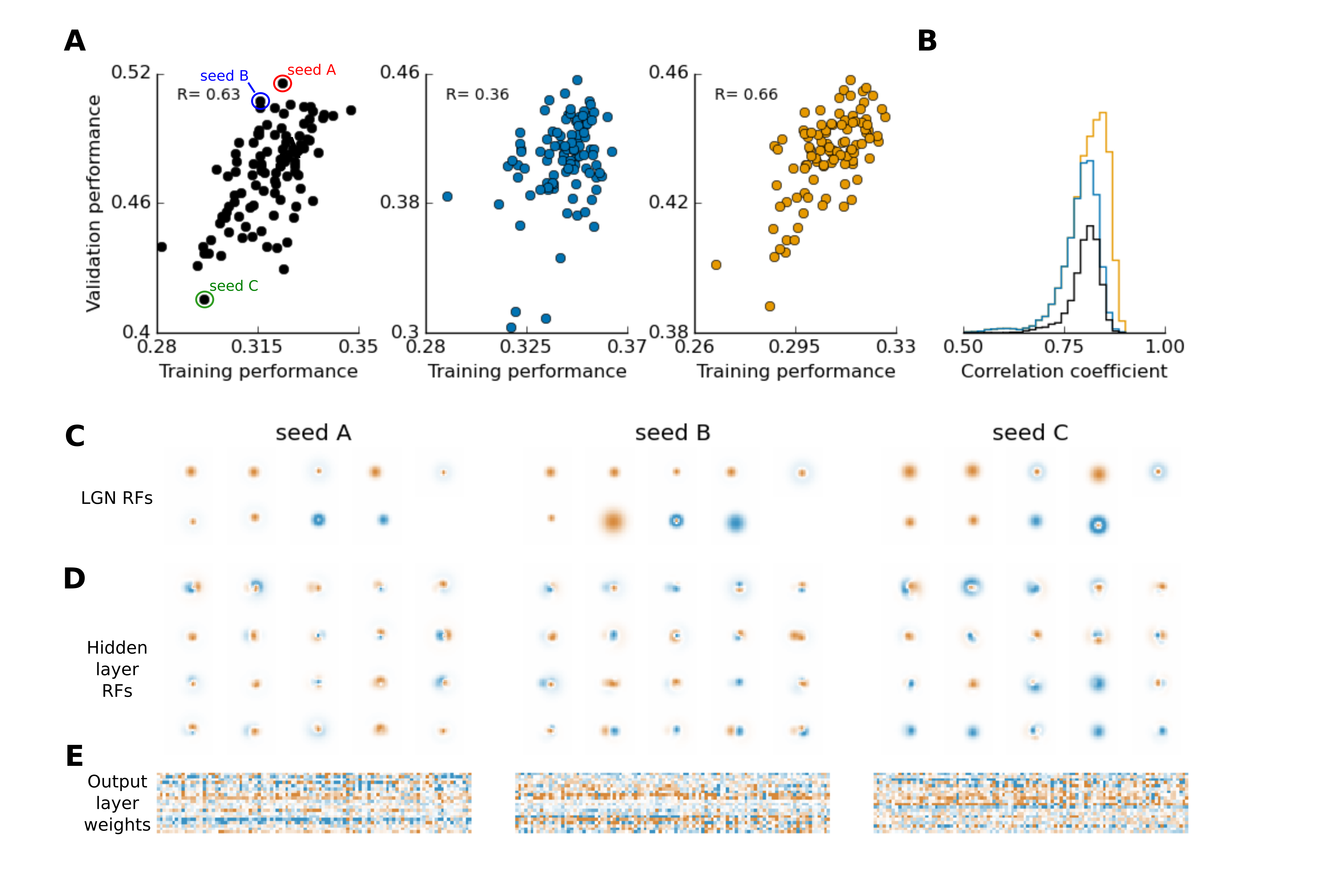

Supplement: S1 Fig — (A) The model performance on the training vs. validation data set across 100 HSM fits using different initial conditions. The three plots show results for each of the 3 imaged regions separately. The color coding of the regions is the same as throughout the main paper. (B) The correlations between responses of pairs of HSM model fits obtained from different initial conditions. (C) The RFs of matched LGN units of three different fits of the HSM model using different initial conditions (the selected initial conditions are marked in A as seed A,B and C). (D) Matched hidden unit RFs. The ordering of the LGN and hidden units in the HSM is arbitrary which complicates comparison of fitted parameters from different initial conditions. When comparing two model parameterizations, in an ideal case, we would like to find a permutation of the LGN and hidden units that maximizes the similarity (for example measured as the mean correlation across corresponding units) between the two sets of units. Finding such permutation is however intractable. Here we have employed simple greedy strategy to match the two sets of units. In C and D the units from seed B were matched to units from seed A and independently the units from seed C were matched to units from seed A. Furthermore, there is redundancy in the HSM model between the polarity of the LGN units and the weights from the LGN units to hidden units, which are not required to be only positive. For this reason the matching of the LGN units is based on the absolute values of their correlations, and for the visualization the LGN units are flipped such that their polarity matches. (E) The weights from hidden to output units. (TIF) [file pcbi.1004927.s003.tif]

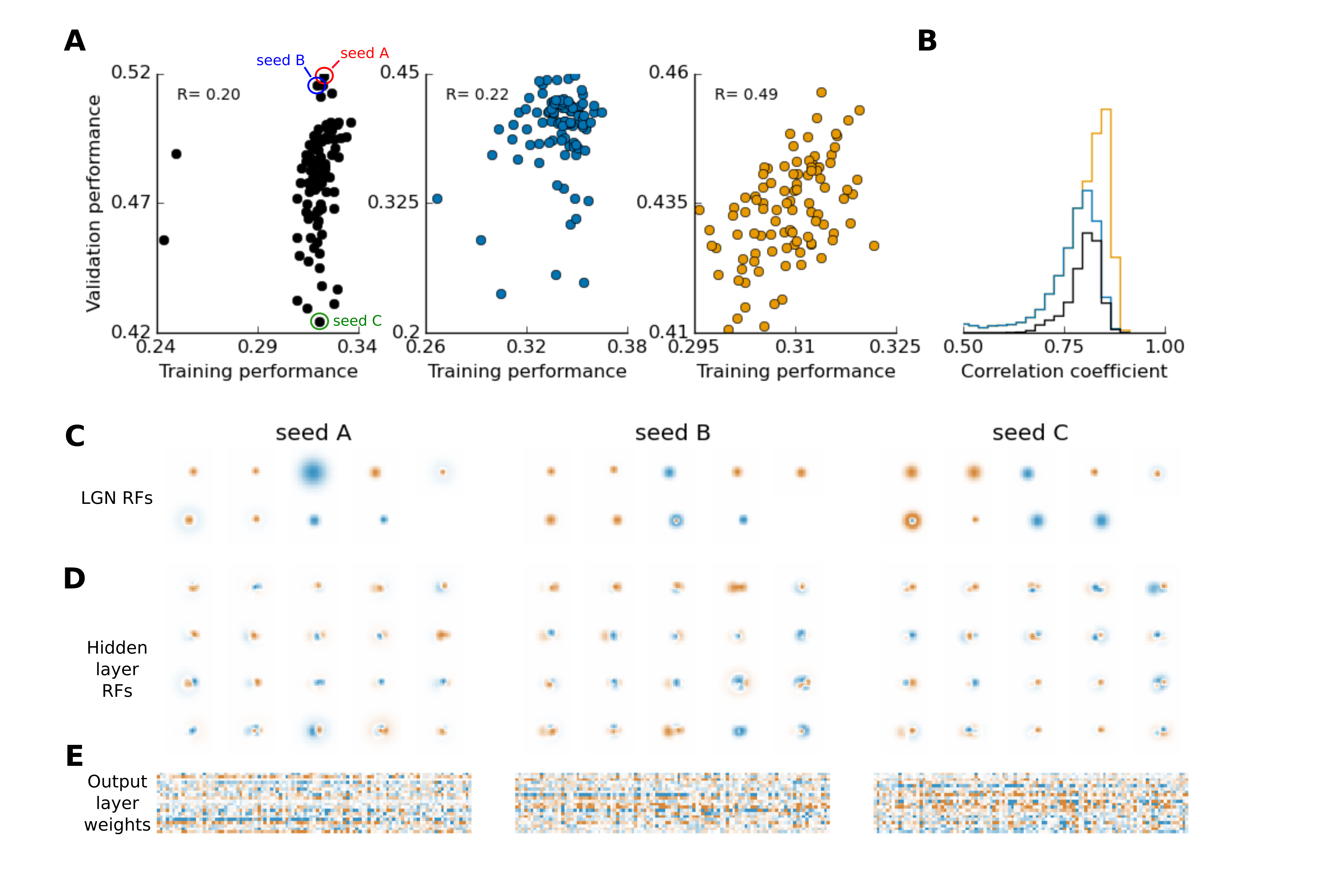

Supplement: S2 Fig — (A) The model performance on training vs. validation data set across 100 HSM fits using different sub-samples of the training set. Each sample was obtained by removing 100 random training stimuli. The three plots show results for each of the 3 imaged regions separately. The color coding of the regions is the same as throughout the main paper. (B) The correlations between responses of pairs of HSM model fits obtained from different training set samples. (C) The RFs of matched LGN units of three fits of HSM to three different samples of training set (the selected samples are marked in A as seed A,B and C). (D) Matched hidden unit RFs. See panel D of S1 Fig caption for details about the matching procedure. (E) The weights from hidden to output units. (TIF) [file pcbi.1004927.s004.tif]
